# Isolated Distal Fibular Stress Fracture after Total Hip Arthroplasty in a Patient with Developmental Dysplasia of the Hip

**DOI:** 10.1155/2020/4218719

**Published:** 2020-01-20

**Authors:** Tomofumi Nishino, Fumi Ochiai, Tomohiro Yoshizawa, Hajime Mishima, Masashi Yamazaki

**Affiliations:** Department of Orthopaedic Surgery, Faculty of Medicine, University of Tsukuba, Japan

## Abstract

Stress fractures following total hip arthroplasty in the lower limbs away from the surgical area are very rare. We report a case of stress fracture in the isolated distal fibula that presented five months after total hip arthroplasty in a patient with developmental dysplasia of the hip. A 67-year-old woman diagnosed with coxarthrosis of the right hip joint, classified as Crowe's group 3, underwent total hip arthroplasty with acetabular reconstruction using a bulk bone graft. The surgery successfully treated the preoperative leg length discrepancy and flexion and external rotation contractures. The alignment of the right lower limbs changed from slight varus to valgus knee following surgery. The postoperative process went well; however, she experienced lateral ankle pain on the affected side five months after surgery. No obvious fracture was observed via radiograph; however, she received a subsequent diagnosis of isolated distal fibula stress fracture. Additionally, she was diagnosed with vitamin D deficiency. Valgus alignment change of the knee joint and vitamin D deficiency were considered the main causes of the stress fracture. Stress fractures should be suspected in patients complaining of unexpected pain following total hip arthroplasty, even in distant areas of the affected limb, especially in osteoporotic patients.

## 1. Introduction

Unidentified fractures around the hip joint following total hip arthroplasty are occasionally found. The intraoperative procedure and the difference in stiffness between the implant and bone are considered the main causes of them. Fracture in the lower limbs, separate from the implant, is very rare. We report an unprecedented case of isolated distal fibular transverse fracture five months after total hip arthroplasty without any history of trauma. We investigated the etiology, with a special focus on the lower limb alignment change before and after surgery, and the bone quality.

## 2. Case Presentation

**Figure 1:**
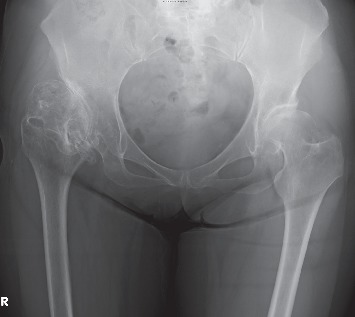
Anteroposterior radiograph of the both hip joints.

**Figure 2:**
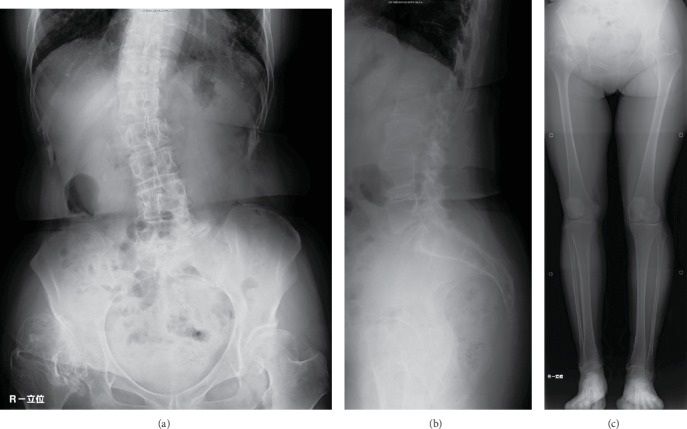
Anteroposterior (a) and lateral (b) radiograph of lumbar spine in standing. Anteroposterior radiograph of bilateral whole lower limbs in standing (c).

A 67-year-old woman was diagnosed with secondary coxarthrosis due to developmental dysplasia of the hip with high hip center. She presented to our hospital for right coxalgia and gait disability due to leg length discrepancy. The contralateral side of her hip joint showed radiographical osteoarthritic change with acetabular dysplasia; however, the symptoms were mild. She was on an aspirin regimen due to a past history of angina. Her height, body weight, and body mass index were 161 cm, 61 kg, 23.5 kg/m^2^, respectively. She was limping with a T-cane, and her activities of daily living were considerably limited because of right coxalgia. Flexion and external rotation contractures were notable in the right hip joint (Table 1), and the right leg length was 2 cm shorter than the left side by measuring the spinal malleolar distance. A radiograph of the hip joint showed right subluxated osteoarthritis classified as Crowe's group 3 [1] (Figure 1). Scoliosis with the convex to the right topped the L1 vertebrae, and a Cobb angle of 22 degrees was measured via standing lumbar anteroposterior radiograph (Figure 2(a)). Flexion contracture of the hip joint caused an increase in lumbar lordosis, and the pelvis was anteverted in the lateral lumbar to pelvic radiograph (Figure 2(b)). The femorotibial angle (FTA) was used to determine the standing bilateral lower limb alignment; the right and left legs measured 178 and 173 degrees, respectively (Figure 2(c)). The values of dual-energy X-ray absorptiometry (DEXA) are shown in Table 2; the right femoral neck decreased slightly, but the others were in the normal range. Laboratory findings were C-reactive protein (CRP): 0.04 (normal range: 0-0.5 mg/dl); alkaline phosphatase: 196 (40-150 IU/l); calcium: 9.6 (8.4-10.2 mg/dl); phosphorus: 3.6 (2.3-4.7 mg/dl); and albumin (3.9-4.9 g/d). Bone turnover markers were tartrate-resistant acid phosphatase 5b (TRACP-5b), 516 (premenopausal normal range: 120-420 mU/dl) and total procollagen type 1 N-terminal propeptide (total P1NP), 45.5 (26.4~98.2 ng/ml). She had no fracture episodes, including fragility fractures.

**Figure 3:**
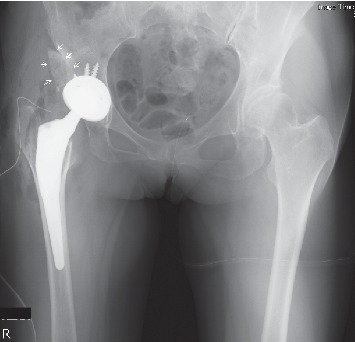
Anteroposterior radiograph immediately after total hip arthroplasty. A bulk bone was grafted by bioabsorbable screws.

Cementless total hip arthroplasty with acetabular reconstruction using a bulk bone graft was performed in this case (Continuum Acetabular System Trabecular Metal Shell, Biolox Delta Modular Ceramic Head, Taperloc Complete Primary Femoral Porous Coated Stem Reduced Distal High Offset type: Zimmer Biomet, TN, USA) (Figure 3). The center of the femoral head moved inferiorly and medially (21 and 45 mm, respectively) postoperation. The leg length discrepancy was resolved in radiographic measurement. Full weight bearing was allowed immediately after surgery as postoperative therapy. Her postoperative process was successful; she could ambulate without a cane three months after the surgery.

**Figure 4:**
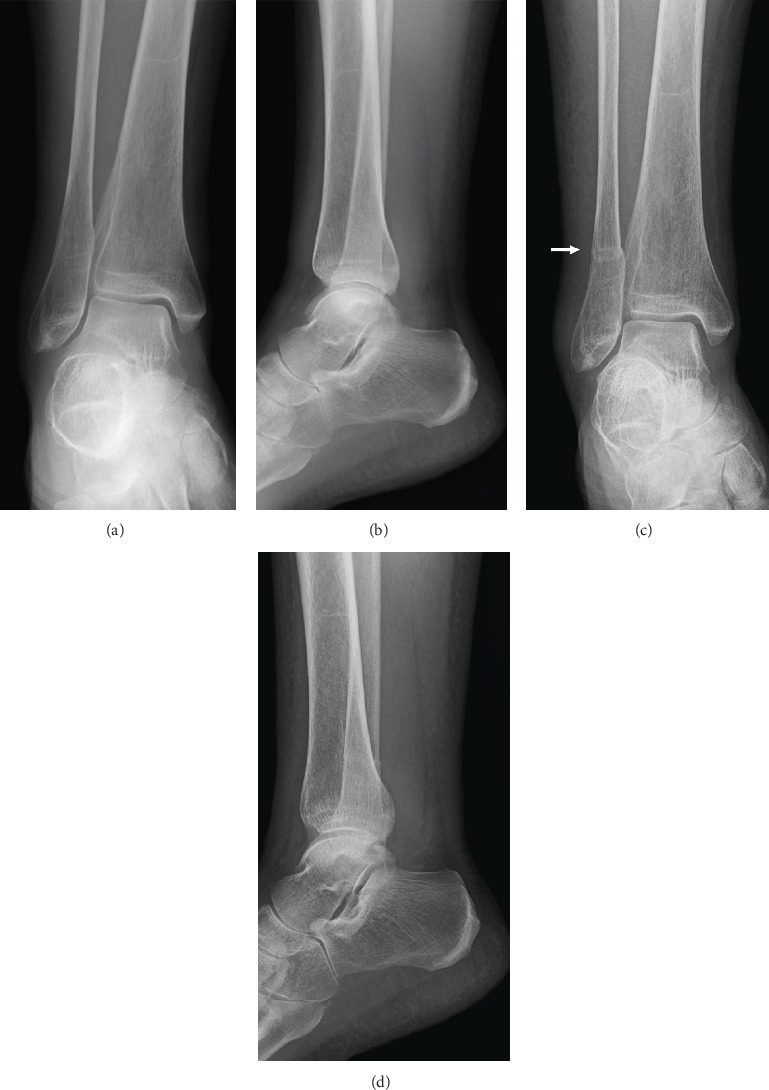
Bilateral radiograph of the right ankle joint immediately after presence of symptoms (a, b) and three weeks after that (c, d). Transverse liner fracture was obvious in (c) (arrow), but not in (a).

**Figure 5:**
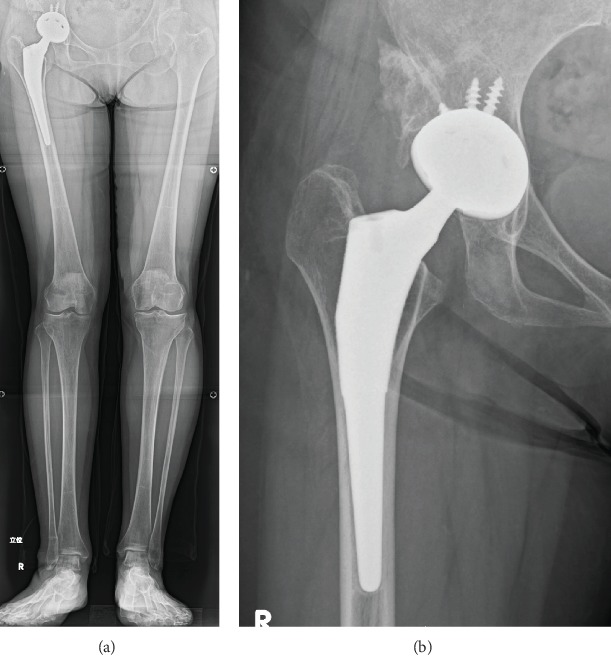
Anteroposterior radiograph of bilateral whole lower limbs in standing (a) and right hip joint (b) six months after surgery.

**Figure 6:**
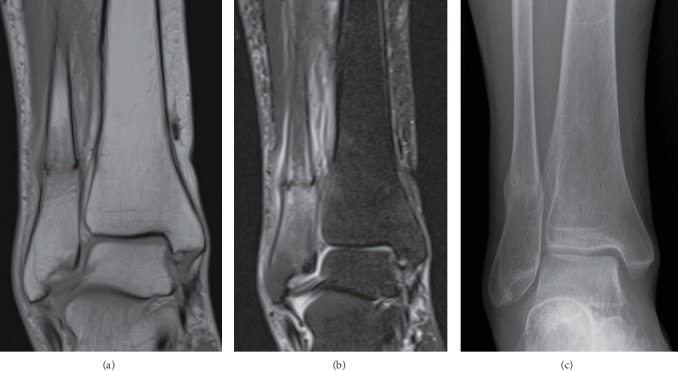
MRI showed an isolated fibular fracture, bone marrow edema, and surrounding soft tissue edema without any pathological lesion: (a) PDW coronal, (b) STIR coronal. Bone union was confirmed in anteroposterior radiograph (c) three months after identifying fracture.

However, she presented with right lateral ankle pain during ambulation and edema of the right foot, without any episode of trauma five months after surgery. A few days later, she visited a different hospital for her symptoms and a radiograph of the right ankle joint was obtained, which did not reveal an obvious fracture (Figures 4(a) and 4(b)). The symptoms persisted and she visited our hospital six months after the surgery. A physical examination revealed localized swelling and tenderness around the right lateral malleolus. A radiograph of the right ankle joint revealed a transverse fracture 5 cm proximal to the lateral malleolus tip (Figures 4(c) and 4(d)). Right and left standing lower limb alignments measured 168 and 176 degrees, respectively, via FTA (Figure 5(a)). A loosening of the implant was not obvious and the grafted bulk bone appeared to union in the radiograph of the hip joint (Figure 5(b)). MRI of the ankle joint showed bone marrow edema of the fibula and soft tissue edema. There were no findings of fracture and edema in the tibia (Figure 6(a) and 6(b)). These image results demonstrated an isolated distal fibular fracture. Regarding the range of motion six months after the surgery, flexion and external rotation contractures improved obviously compared to the preoperative value (Table 1). The value of the 25(OH) vitamin D was 9.0 ng/dl, and she was diagnosed with vitamin D deficiency. We considered this fracture a result of the increased stress concentrated on the distal fibular from the lower limb alignment change following the total hip arthroplasty. Factors of fragility fracture, such as vitamin D deficiency and local osteoporosis from disuse, might also contribute to this etiology. We administered eldecalcitol 0.75 *μ*g per day orally for vitamin D deficiency and a U-shape splint for fracture. Weight bearing was allowed with splint and T-cane use. Bone union was confirmed on a follow-up radiograph three months after splinting (Figure 6(c)). The resultant recovery process was favorable.

Informed consent to be published for publication was obtained from the patient, and this study was approved by our institution's ethics review board.

## 3. Discussion

Stress fractures are the result of repetitive mechanical stress to the bone and may occur in regions of normal and abnormal bones. Pentecost et al. classified them into three groups based on the quality of the bone: fatigue fractures, which appear in a bone with normal strength; insufficiency fractures, which appear in fragile osteoporotic bones; and pathologic fractures, which appear in fragile bones as a result of tumor invasion [2]. Regarding the postoperative alignment change, the right hip joint was classified as Crow's group 3 subluxated hip, where the center of the femoral head was positioned higher and more lateral than the original position. Therefore, there was a leg length discrepancy, scoliosis, and hyperlordosis of the lumbar spine, and mild varus deformity of the right knee joint. The surgery moved the center of the femoral head inferiorly and medially, almost to its original position. Decreasing the offset and the external rotation contracture decreased the FTA by 10 degrees and changed the right knee joint to valgus alignment. We presume the alignment change made the mechanical axis move laterally, causing a concentrated mechanical stress of eversion on the distal fibula. Lower limb alignment might be affected in patients who have hip osteoarthrosis with leg length discrepancy for an extended period. Previous studies confirmed that the knee contralateral to the affected hip tends to be varus and the ipsilateral knee tends to be valgus, similar to the phenomenon known as “windswept deformity” [3, 4]. In this case, slight varus deformity was presented in the affected lower limb preoperatively against consideration of the statement above. It is supposed that it participated the dysplasia of the hip in the contralateral side and lumbar scoliosis existed considering alignment change in the frontal plane. As a result, it changed into valgus deformity.

As for bone quality in this case, the patient had no obvious osteoporosis per lumbar spine and femoral neck values by DEXA; however, local disuse osteoporosis of the affected limb was suggested. Furthermore, the vitamin D deficiency, cleared after the fracture, might be associated with this etiology. Therefore, this was considered to be an insufficiency fracture in stress fracture using Pentecost et al.'s classification.

It is known that fractures may occur without a significant cause after total hip arthroplasty. Occult fracture made by surgeons during operation, especially during the insertion of the implant into the acetabular and femur, is a main cause of fractures. The difference in stiffness between the inserted implant material (usually made by cobalt chrome or titanium) and the bone is considered another main cause of them. Therefore, they occur around the hip joint, pelvis, and/or proximal femur, where the surgery was performed [5, 6]. Fractures in the lower limbs, apart from the surgical areas, are rare; however, proximal tibia [7], distal tibia and fibula [8], and calcaneus [9] fractures have been previously reported. This is a first report of an isolated distal fibula fracture following arthroplasty. In previous reports, clinical symptoms appeared ten weeks to six months after surgery, similar to our case. There are few reports of isolated fibula stress fractures unassociated with arthroplasty. Burrows et al. were able to segregate two groups in which stress fractures occurred in the lowest third of the fibula. The first group was comprised of young athletes, such as runners and skaters. It is not difficult to understand the mechanism of fracture or fractures in such activities. The second group is made up of women of middle age or older who are often on their feet over the course of the day. There are probably several etiological factors in this second group [10]. The patient in this case did not use her foot much; however, the relative load to the foot was increased, as mentioned above. Therefore, our case belongs in this second group. The fracture line is transverse, as in other stress fractures. The site of the fractures seems to offer an explanation of the mode of production. In our case, they were situated 5 cm above the tip of the lateral malleolus; a similar level was noted by Burrows et al. Here, the bone is largely cancellous, and the fracture line is located at a gap between the interosseus ligament and syndesmosis. Continued eversion of the feet strains the lateral aspect of the fibula. Cheng et al. reported a stress fracture of the distal fibula in a flatfoot patient [11]. They concluded excessive load to the distal fibula from the flatfoot deformity caused the fracture. In our case, there was no foot deformity or abnormality of the leg-heel angle.

Early diagnosis of stress fractures is difficult because of their rarity, and early radiographs reveal no obvious fracture. A bone scintigram is reported to be useful for the early diagnosis of stress fractures, whereas computed tomography scanning is of little value in the diagnosis of stress fractures of the long bones [12]. MRI is the first choice for diagnosis of stress fracture of lower limbs from various points of view [13]. It is also useful for insufficiency fractures in the spine and pelvis which have diagnostic difficulty. [14] The proper diagnosis of our case was made over the course of one month. In the absence of MRI, it is necessary to recognize such an etiology. Repeated radiography is recommended when stress fractures are suspected. Orthopaedic surgeons should be aware of the potential for foot and ankle stress fractures following total hip arthroplasty, especially in osteoporotic women.

## 4. Conclusion

We reported a case of isolated distal fibular stress fracture following total hip arthroplasty. The alignment changed after surgery, and the bone quality of the patient was associated with this fracture etiology. Stress fractures should be suspected in patients complaining of unexpected pain following total hip arthroplasty, even in distant areas of the affected limb, especially in osteoporotic patients.

## Figures and Tables

**Table 1:** Range of motion before and after surgery in the right hip joint of the patient.

	Before surgery (degree)	6 months after surgery (degree)
Flexion	90	90
Extension	-10	0
Abduction	15	30
Adduction	10	15
External rotation	45	45
Internal rotation	-5	45

**Table 2:** Bone mineral density before surgery.

	Bone mineral density (g/cm^2^)	*T*-score	*Z*-score
Lumber spine	0.830	-1.6	0.4
Right femoral neck	0.593	-1.8	0.0
Left femoral neck	0.660	-1.2	0.8
